# ALCOHOL USE DISORDER AND DEPRESSION IN PATIENTS AFTER UNDERGOING BARIATRIC SURGERY

**DOI:** 10.1590/0102-6720202500002e1871

**Published:** 2025-03-14

**Authors:** Kátia Cristina OLIVEIRA, Fernando SANTA-CRUZ, Luciana Melo Souza LEÃO, Flávio KREIMER, Álvaro Antonio Bandeira FERRAZ

**Affiliations:** 1 Universidade Federal de Pernambuco, Postgraduate in Surgery - Recife (PE), Brazil; 2 Hospital dos Servidores do Estado, General Surgery Service - Recife (PE), Brazil; 3 Universidade Federal de Pernambuco, Hospital das Clínicas, Department of Psychology - Recife (PE), Brazil; 4 Universidade Federal de Pernambuco, Hospital das Clínicas, General Surgery Service - Recife (PE), Brazil

**Keywords:** Alcoholism, Depression, Obesity, Bariatric Surgery, Alcoolismo, Depressão, Obesidade, Cirurgia Bariátrica

## Abstract

**BACKGROUND:**

Research indicates that patients undergoing bariatric surgery face a six to seven times higher risk of developing alcohol use disorder (AUD) compared with the population of obese individuals not undergoing surgical intervention. Studies suggest that problematic alcohol consumption encompassing depression escalates gradually after surgery.

**AIMS:**

The purpose of this study was to evaluate the impact of bariatric surgery on the incidence of AUD and depression during the postoperative period.

**METHODS:**

Prospective study that evaluated 68 patients who underwent either sleeve gastrectomy (SG) or Roux-en-Y gastric bypass (RYGB). The presence of AUD and depression was assessed both pre- and post-operatively. AUD assessment utilized the AUD identification test-C score, whereas depression assessment employed the Beck Depression Inventory (BDI).

**RESULTS:**

The average age of the sample was 42.81±9.28 years, with 85.3% being female. The mean follow-up was 16.54±7.41 months. In the preoperative assessment, 92.6% of the sample fell into the low-risk category for AUD. No significant difference was observed between the RYGB and SG groups. Postoperatively, 89.7% of the sample was classified as low risk for AUD, with no significant differences compared with the preoperative assessment. Regarding depression, there was no significant difference between pre- and post-operative periods for all patients. However, a notable trend toward a reduction in “severe depression” was observed in the postoperative period for patients undergoing SG (pre: 14.0% vs. post: 7.0%, p=0.013).

**CONCLUSIONS:**

There is no significant difference in the presence of AUD and depression between pre- and post-operative assessments in patients who have undergone bariatric surgery.

## RESUMO

**Racional:**

Pesquisas indicam que pacientes submetidos à cirurgia bariátrica enfrentam um risco seis a sete vezes maior de desenvolver transtorno por uso de álcool (TUA) em comparação à população de indivíduos obesos que não passam por intervenção cirúrgica. Estudos sugerem que o consumo problemático de álcool, incluindo depressão, aumenta gradualmente após a cirurgia.

**OBJETIVOS:**

Avaliar o impacto da cirurgia bariátrica na incidência de transtorno do uso de álcool (TUA) e depressão durante o período pós-operatório.

**MÉTODOS:**

Estudo prospectivo avaliando 68 pacientes submetidos à gastrectomia vertical (GV) ou bypass gástrico em Y-de-Roux (BGYR). A presença de TUA e depressão foi avaliada tanto no pré quanto no pós-operatório. A avaliação de TUA utilizou o escore AUDIT-C, enquanto a depressão foi avaliada pelo Inventário de Depressão de Beck (IDB).

**RESULTADOS:**

A idade média da amostra foi de 42,81±9,28 anos, sendo 85,3% do sexo feminino. O acompanhamento médio foi de 16,54±7,41 meses. Na avaliação pré-operatória, 92,6% da amostra estava na categoria de baixo risco para TUA. Não houve diferença significativa entre os grupos BGYR e GV. No pós-operatório, 89,7% da amostra foi classificada como de baixo risco para TUA, sem diferenças significativas em relação à avaliação pré-operatória. Em relação à depressão, não houve diferença significativa entre os períodos pré e pós-operatório para todos os pacientes. No entanto, foi observada uma tendência significativa de redução da “depressão severa” no período pós-operatório para os pacientes submetidos à GV (pré: 14,0% vs. pós: 7,0%, p=0,013, p<0.05).

**CONCLUSÕES:**

Não há diferença significativa na presença de transtorno do uso de álcool e depressão entre as avaliações pré e pós-operatórias em pacientes submetidos à cirurgia bariátrica.

## INTRODUCTION

In Brazil, the prevalence of obesity has surged over time, escalating from 11.6% in 2006 to 22.4% in 2021^19^ ^,^ ^26^ ^,^ ^35^ ^,^ ^36^. In instances of severe obesity, bariatric surgery (BS) is deemed the optimal intervention for weight loss^27^, aiding in the management and reduction of associated diseases and their consequences^3^ ^,^ ^8^ ^-^ ^10^ ^,^ ^31^ ^,^ ^35^ ^,^ ^36^. Studies underscore that 7 to 33% of patients undergoing BS may develop alcohol use disorder (AUD)^4^ ^,^ ^11^ ^,^ ^18^ ^,^ ^22^.

Research indicates that patients undergoing BS face a six to seven times higher risk of developing AUD compared with the population of obese individuals not undergoing surgical intervention^30^. Temporally, studies suggest that problematic alcohol consumption escalates gradually after surgery and is particularly pronounced in patients 3-4 years post-BS^25^.

Several explanations regarding the heightened risk of AUD post-BS have been posited. One pertains to the physiological changes post-BS, wherein there is an alteration in alcohol metabolism, resulting in substantially elevated peaks of alcohol in venous blood, alongside a prolonged return to sober levels^1^ ^,^ ^12^ ^,^ ^32^.

Additionally, neurobiological contributions substantiate that drugs including alcohol elicit a rewarding effect by activating dopaminergic neurons in the ventral tegmental area, leading to dopamine release in the nucleus accumbens. Food addiction, encompassing various hypothalamic neuropeptides regulating food intake, such as leptin, insulin, ghrelin, orexin, cholecystokinin, peptide YY, and neuropeptide Y, further complicates the scenario^13^ ^,^ ^16^. Recent evidence underscores the pivotal role of orexin not only in the dysregulation of eating behavior but also in the recruitment of the orexin neuronal circuit by drugs of abuse, accentuating the convergence of reward processes within the hormonal system^12^.

Given the landscape, it becomes imperative to conduct prospective studies that comprehensively delineate the association between BS and neuropsychiatric disorders, encompassing AUD and depression^15^ ^,^ ^34^. 

This study seeks to assess the impact of Roux-en-Y gastric bypass (RYGB) and sleeve gastrectomy (SG) on the incidence of AUD and depression during the postoperative period in patients who have undergone BS.

## METHODS

The prospective cohort study was conducted at the Hospital das Clínicas of the Universidade Federal de Pernambuco with the aim of evaluating alcohol consumption patterns and the prevalence of depressive disorders in the pre- and late post-operative periods following BS. Inclusion criteria encompassed patients of both genders aged between 18 and 64 years, possessing a body mass index (BMI) of 35 kg/m^2^ and having an indication for BS using the RYGB or SG techniques. Patients with a follow-up duration of less than 6 months were excluded from the analysis.

Patients underwent interviews in both the preoperative period and 6 months postsurgery, during which demographic data were collected to characterize the sample. Two questionnaires, namely the AUD identification test (AUDIT-C) and the Beck Depression Inventory (BDI), were administered to assess alcohol consumption patterns and the presence of depressive disorders, respectively. These questionnaires were applied both preoperatively and 6 months postoperatively.

The AUDIT-C evaluates varying levels of alcohol use, ranging from nonuse to probable dependence, considering consumption patterns over the preceding 12 months^5^ ^,^ ^23^ ^,^ ^29^. It classifies as “low-risk use” situations where the likelihood of alcohol-related problems is minimal, designates “risky use” when consumption may compromise health, and identifies “harmful use” as a condition where alcohol consumption heightens the risk of developing an AUD or suffering from dependence (Table 1).

**Table 1:** AUDIT-C score.

Scoring system						
Questions	0	1	2	3	4	Your score
How often do you have a drink containing alcohol?						
How many units of alcohol do you drink on a typical day when you are drinking?						
How often have you had six or more units if female, or eight or more if male, on a single occasion in the last year?						
AUDIT-C score						

0-4: low risk; 5-7: increasing risk; 8-10: high risk; 11-12: possible dependence.

The BDI gauges the severity of depressive episodes, categorizing them as mild, moderate, or severe^6^ ^,^ ^7^ ^,^ ^17^. Comprising 21 questions about the individual’s feelings in the past week, each question features four items with diverse intensity choices. For instance:

0. I don’t feel sad,
1. I feel sad;
2. I feel sad all the time and can’t get out of this situation; and
3. I feel so sad or unhappy that I cannot bear it.

The total scoring is considered for final conclusions.

All procedures performed in this study involving human participants were in accordance with the ethical standards of the institutional research committee and the 1964 Helsinki Declaration and its later amendments. This research project was approved by the Ethics Committee of our institution (number 2.947.845).

### Statistical analysis

Comparisons between types of surgery or groups for categorical variables employed Pearson’s chi-square test or Fisher’s exact test when the conditions for using the chi-square test were not met. The Kruskal-Wallis test was applied to compare the groups in ordinal variables. Assessment comparisons utilized the paired Wilcoxon test for ordinal variables, and the McNemar test was used for categorical variables. Normality was assessed using the Shapiro-Wilk test, and equality of variances was verified using Levene F tests. A significance level of 5% was adopted for statistical tests, and data entry was performed using an Excel spreadsheet. Statistical calculations were conducted using IBM SPSS version 25.

## RESULTS

The final analysis included a total of 68 patients, with 43 in the SG group and 25 in the RYGB group. Table 2 shows that the mean age was slightly over 2 years higher in the SG group compared with the RYGB group (43.62 vs. 41.44 years), and the mean time between surgery and the interview was approximately 2 months longer in the RYGB group than in the SG group (17.85 vs. 15.77 months). Nevertheless, no significant differences (p>0.05) were observed between the two types of surgery for either of the two variables analyzed. There were no significant differences concerning the presence of hypertension and diabetes in the sample during the preoperative period (baseline).

**Table 2:** Statistics of numerical variables in the total group and according to the type of surgery.

Type of surgery				
Variable	SG (43)	RYGB (25)	Total (68)	p-value
	Mean±SD	Mean±SD	Mean±SD	
Age	43.62±8.20	41.44±10.92	42.81±9.28	p=0.357*
Female	37 (86.0)	21 (84.0)	58 (85.3)	p=1.000*
Diabetes	12 (27.9)	10 (40.0)	22 (32.4)	p=0.304*
HBP	29 (67.4)	15 (60.0)	44 (64.7)	p=0.536*
Follow-up	15.77±5.53	17.85±9.85	16.54±7.41	p=0.937^†^

*Student t-test with equal variances; ^†^Mann-Whitney test.

SG: sleeve gastrectomy; RYGB: Roux-en-Y gastric bypass; SD: standard deviation; HBP: high blood pressure.

Regarding associated diseases, the most prevalent conditions in the preoperative evaluation were anxiety, with a higher percentage in the RYGB group than in the SG group (88.0 vs. 69.8%), arterial hypertension, with 67.4% in the SG group and 60.0% in the RYGB group, and diabetes, higher in the RYGB group (40.0 vs. 27.9%), and depression (20.9% in the SG group and 20.0% in the RYGB group). In the postevaluation, the majority experienced anxiety, with 65.1% in the SG group and 56.0% in the RYGB group; the frequencies of associated diseases (hypertension, diabetes, depression, and anxiety) were lower than before in both groups. The most substantial reductions occurred for arterial hypertension, which decreased significantly (in SG from 67.4 to 7.0% and in RYGB from 60.0 to 4.0%), in diabetes (in SG from 27.9 to 4.7%, and in RYGB from 40.0 to 4.0%), and in anxiety in the RYGB group, which decreased from 88.0 to 56.0% (Table 3).

**Table 3:** Assessment of associated pathologies.

Type of surgery				
Comorbidity	SG (43)	RYGB (25)	Total (68)	p-value
	n (%)	n (%)	n (%)	
Pre-diabetes	12 (27.9)	10 (40.0)	22 (32.4)	p=0.304*
Post-diabetes	2 (4.7)	1 (4.0)	3 (4.4)	p=1.000^†^
p-value	p=0.002^‡,§,//^	p=0.004^‡,§,//^	p<0.001^‡,§,//^	
Pre-HBP	29 (67.4)	15 (60.0)	44 (64.7)	p=0.536*
Post-HBP	3 (7.0)	1 (4.0)	4 (5.9)	p=1.000^†^
p-value	p<0.001^‡,§^	p<0.001^‡,§^	p<0.001^‡,§^	
Pre-depression	9 (20.9)	5 (20.0)	14 (20.6)	p=0.927*
Post-depression	5 (11.6)	3 (12.0)	8 (11.8)	p=1.000^†^
p-value	p=0.219^‡^	p=0.625^‡^	p=0.109^†^	
Pre-anxiety	30 (69.8)	22 (88.0)	52 (76.5)	p=0.087*
Post-anxiety	28 (65.1)	14 (56.0)	42 (61.8)	p=0.456*
p-value	p=0.774^‡^	p=0.021^‡,§^	p=0.052^‡,§^	

*Pearson chi-square test; ^†^Fisher exact tests; ^‡^McNemar test; ^§^Significant difference at 5%; ^//^Paired Wilcoxon test.

SG: sleeve gastrectomy; RYGB: Roux-en-Y gastric bypass; HBP: high blood pressure.

Table 4 shows the results of the AUDIT-C and BDI scales by group and assessment. According to this table, the majority in each type of surgery and each evaluation was classified by AUDIT as low risk, with percentages ranging from 84.0 to 93.3%, followed by the increased risk category, with values ranging from 2.3 to 12.0%. No significant differences were observed between the types of surgery in each evaluation, nor between the types of evaluation in each type of surgery. The majority in each type of surgery and each evaluation were classified as having no depression. The percentages of that category increased from pre- to post-surgery (from 53.3 to 76.7% among those undergoing SG surgery and from 68.0 to 76.0% in the RYGB group), except for the percentage of cases with severe depression in the RYGB group, which increased from one (4.0%) to three (12.0%) patients from pre- to post-surgery. In the mild depression and moderate depression categories, there was a reduction from pre- to post-periods. In the other categories, there was a reduction in depression. In the type of SG surgery, cases of mild, moderate, and severe depression reduced from pre- to post-surgery from 16.3, 16.3, and 14.0% to 4.7, 11.6, and 7.0%, respectively. In the RYGB surgery, the percentages of cases with mild and moderate depression reduced from pre- to post-periods from 20.0 and 8.0 to 12.0% and zero, respectively. The only significant difference (p<0.05) was found between the two assessments in the SG group.

**Table 4:** Assessment of Alcohol Use Disorders Identification Test and Beck Depression Inventory by evaluation in the total group and according to the type of surgery.

Type of surgery					
Variable	SG	RYGB	Total	p-value
	n (%)	n (%)	n (%)	
Pre-AUDIT-C				
Low risk	41 (95.3)	22 (88.0)	63 (92.6)	p=0.255*
Increasing risk	2 (4.7)	2 (8.0)	4 (5.9)	
Higher risk	-	1 (4.0)	1 (1.5)	
Post-AUDIT-C				
Low risk	40 (93.0)	21 (84.0)	61 (89.7)	p=0.267*
Increasing risk	1 (2.3)	3 (12.0)	4 (5.9)	
Higher risk	2 (4.7)	1 (4.0)	3 (4.4)	
p-value	p=0.500^†^	p=1.000^†^	p=0.312^†^	
Pre-BDI				
Mild depression	7 (16.3)	5 (20.0)	12 (17.6)	p=0.142*
Moderate depression	7 (16.3)	2 (8.0)	9 (13.2)	
Severe depression	6 (14.0)	1 (4.0)	7 (10.3)	
Post-BDI				
Mild depression	2 (4.7)	3 (12.0)	5 (7.4)	p=0.966*
Moderate depression	5 (11.6)	-	5 (7.4)	
Severe depression	3 (7.0)	3 (12.0)	6 (8.8)	
p-value	p=0.013^†,‡^	p=1.000^†^	p=0.052^†^	
Total	43 (100.0)	25 (100.0)	68 (100.0)	

*Mann-Whitney test; ^†^paired Wilcoxon test; ^‡^Significant difference at 5%.

SG: sleeve gastrectomy; RYGB: Roux-en-Y gastric bypass; AUDIT-C: Alcohol Use Disorders Identification Test; BDI: Beck Depression Inventory.

## DISCUSSION

The primary objective of the current study was to examine the impact of BS on the incidence of AUD and depression throughout the postoperative period. The results indicate that there was no significant increase in the incidence of these disorders following BS. Similarly, there was no statistically significant difference between the studied surgical techniques (RYGB vs. SG), with an AUD incidence of 4.0% after RYGB and 4.7% after SG, with an average follow-up of 16 months.

AUD is acknowledged as an undesired consequence of metabolic BS, potentially arising from modifications in alcohol metabolism, pharmacokinetics, reward processing, or the transfer of dependence after surgery^14^. The seminal studies of the Swedish Obese Subjects Study (SOS), conducted in 2013, revealed that 93.1% of operated patients reported alcohol consumption classified as low risk by the World Health Organization (WHO)^33^. Our findings align with these results, indicating that 92.6% of surveyed patients exhibited a “low-risk” alcohol consumption pattern, indicative of a lower probability of developing alcohol-related problems.

A prospective study by Ibrahim et al.^18^ demonstrated that patients undergoing SG face a similar risk of developing AUD compared with those undergoing RYGB 2 years after surgery. The association between BS and AUD was also explored in the prospective cohort study Longitudinal Assessment of Bariatric Surgery-2 (LABS-2), revealing a cumulative incidence of AUD ranging from 10 to 21% 2-5 years after surgery^20^. Another longitudinal assessment found that before surgery, more than half of the participants reporting AUD in the preoperative assessment continued to have or experienced recurrent AUD in the first 2 years after surgery. Additionally, 7.9% of participants who did not report AUD in the preoperative evaluation developed AUD in the postoperative period^21^. Furthermore, the study of Mahmud et al.^24^ noted that patients undergoing RYGB have an increased risk of AUD-related hospitalizations compared to those undergoing SG.

However, the data from our study did not reveal a significant difference in the incidence of AUD before and after BS (1.5 vs. 4.4%), nor with respect to the type of BS: RYGB (AUD/harmful use: 4.0%) and SG (AUD/harmful use: 4.7%). There was also no distinction in terms of consumption patterns before and after surgical intervention: low-risk use before (RYGB 88% vs. SG 95.3%) and after surgery (RYGB 84 vs. SG 4.7%), and mild risk after surgery (RYGB 12 vs. SG 2.3%), and harmful use before surgery (RYGB 4 vs. SG 4.7%). The studies mentioned earlier suggest that the development of AUD typically begins to manifest from the first year after surgery and increases over time, a trend not substantiated in our study, underscoring the heterogeneity of findings. Nevertheless, it aligns with the consensus that there are no significant differences in AUD incidence between different types of bariatric interventions.

Research indicates that nearly a third of patients undergoing BS experience depression, with 19.8% classified as mild, 7.5% as moderate, and 3.5% as severe. A significant association has been identified between preoperative depressive symptoms and postsurgical hospital stay, as well as higher rates of smoking and alcohol use among the studied patients studied^16^ ^,^ ^28^. Another study demonstrated that the prevalence of psychiatric disorders, including AUD, recreational drug use, and depression, was higher in the BS group compared with other abdominal surgeries^2^.

Our data revealed that more than 40% of BS candidates presented with depression (mild 17.6%, moderate 13.2%, and severe 10.3%), and post-bariatric intervention, there was a significant decrease (mild 7.4%, moderate 7.4%, and severe 8.8%). However, a significant difference was only observed when comparing before and after SG (14 vs. 7%).

Among the limitations of this study, we acknowledge the mean postoperative follow-up time, which was less than 24 months, and the limited sample size due to the reduction in elective surgeries during the study period. It is noteworthy that this study was conducted amid the COVID-19 pandemic, wherein psychological distress due to this context, coupled with social isolation, not only increased the global prevalence of obesity but also emerged as a significant risk factor for AUD.

Although substantial progress has been made in understanding the pathophysiology of BS, considerable strides are yet to be taken to gain a deeper comprehension of the psychological and psychopathological imbalances resulting from these interventions.

## CONCLUSIONS

Considering the presented results, it can be inferred that employing the methodology applied in the present study, there is no substantial increase in the incidence of AUD following BS, irrespective of the technique employed. Concerning depression, there is a notable decrease in the incidence of cases of mild, moderate, and severe depression after SG. Prospective studies involving a larger cohort and extended follow-up periods are imperative to elucidate comprehensively the impacts of BS on the occurrence of the disorders.

## Central Message

Research indicates that patients undergoing bariatric surgery face a six to seven times higher risk of developing alcohol use disorder (AUD) compared with the population of obese individuals not undergoing surgical intervention. Studies suggest that problematic alcohol consumption escalates gradually after surgery and is particularly pronounced in patients 3-4 years post-bariatric surgery. It becomes imperative to conduct prospective studies that comprehensively delineate the association between bariatric surgery and neuropsychiatric disorders, encompassing AUD and depression.

## Perspectives

Considering the presented results, it can be inferred that employing the methodology applied in the present study, there is no substantial increase in the incidence of alcohol use disorder (AUD) following bariatric surgery, irrespective of the technique employed. Concerning depression, there is a notable decrease in the incidence of cases of mild, moderate, and severe depression after sleeve gastrectomy (SG).
